# Association Between Alexithymia, Social Support, and Duration of Methamphetamine Use Among Male Methamphetamine-Dependent Patients

**DOI:** 10.3389/fpsyt.2021.713210

**Published:** 2021-09-21

**Authors:** Shu Cui, Fangshuo Cheng, Qiuyu Yuan, Ling Zhang, Lei Wang, Kai Zhang, Xiaoqin Zhou

**Affiliations:** ^1^Chaohu Hospital, Anhui Medical University, Hefei, China; ^2^School of Mental Health and Psychological Sciences, Anhui Medical University, Hefei, China; ^3^Department of Mental Health, Fourth Affiliated Hospital of Zhejiang University School of Medicine, Yiwu, China

**Keywords:** methamphetamine, methamphetamine-dependent patients, compulsory detoxification, alexithymia, social support, duration of methamphetamine use

## Abstract

**Introduction:** China has 1.18 million methamphetamine abusers. Among the illegal drugs in China, methamphetamine has the highest abuse rate. Although previous studies have indicated a positive relationship between alexithymia and declining social support, the incidence of alexithymia, the total duration of methamphetamine dependence, social support, and the relationships between them among methamphetamine-dependent patients in the Chinese population have been rarely reported.

**Methods:** A total of 113 methamphetamine-dependent patients (all male, mean age 30.45 ± 3.81 years) were enrolled in this cross-sectional study. General demographic data were collected. Alexithymia and social support were measured by Toronto Alexithymia Scale and Social Support Rating Scale.

**Results:** Duration of methamphetamine use among Chinese male methamphetamine-dependent patients in compulsory detoxification was 8.01 ± 3.80 years on average, 23% (26/113) methamphetamine-dependent patients were considered to have alexithymia personality traits. Compared with short-duration methamphetamine-dependent patients (≤8 years), long-duration methamphetamine-dependent patients (> 8 years) were characterized by older age, higher incidence of alexithymia, less subjective social support and support availability, and greater difficulty in identifying feelings. The results of correlation analysis and multiple linear regression analysis indicated that the total duration of methamphetamine use was positively correlated with difficulty in identifying feelings, but negatively correlated with subjective social support.

**Conclusions:** This study provides support for an association between the duration of methamphetamine use and difficulty in identifying feelings or subjective social support. Although the causality is still unclear, this finding should be considered in the psychotherapy of methamphetamine rehabilitation.

## Introduction

Methamphetamine has a worldwide prevalence rate of illegal drug abuse second only to cannabis (1), and it is also the illegal drug with the largest number of abusers in China. In June 2020, the “Drug Situation in China (2019)” released on the website of the Chinese government shows that among the 2.148 million existing drug abusers in China, 1.186 million abusers suffer from methamphetamine dependence, accounting for 55.2% (2). With the rapid development of economic globalization and social information technology, the problem of methamphetamine abuse has spread around the world. Methamphetamine use has been linked to mental disorders, self-injurious behavior, accidental death, and crime (3–5). Despite the availability of effective detoxification methods, relapse rates of methamphetamine remain high (6). Therefore, it is necessary to study the influencing factors of methamphetamine use duration and reliable relapse predictors, and the development of secondary prevention based on the understanding of relapse is essential.

Individual personality traits and perceived social support are risk factors for methamphetamine use (7, 8), nevertheless the relationship between different personality traits and perceived social support isn't entirely clear. Alexithymia is called affective dyslexia or the inability to express emotions, characterized by difficulties identifying, differentiating, and articulating emotions (9). Alexithymia is not a separate diagnosis of mental illness, but a personality trait. More and more studies emphasize that alexithymia has been identified as a negative predictor of many medical outcomes, including substance use disorder, addictive behaviors, depression, and emergence of suicidal ideation and behaviors (10–16). A previous study found that alexithymia occurred in 57.32 percent of female drug abusers (17). In the practice of drug rehabilitation psychological counseling, researchers (18) have also found that most of the patients in compulsory detoxification centers have alexithymia symptoms, which are manifested as difficulty in identifying and expressing their own and other people's emotions in group psychological counseling, poor introspection ability, and often confusing emotional and physical symptoms (17). De Berardis et al. ‘s study provides a novel perspective to understand this phenomenon. That is, alexithymia can lead to emotional disturbances and dysregulation in adolescents that further increase the risk of addictive behaviors and suicidal thoughts in impending adult life, exacerbating emotional dysregulation, forming a vicious cycle that will be difficult to break (13, 15, 19, 20). Alexithymia not only affects the interpersonal skills of drug abusers but also affects the outcomes of psychological drug rehabilitation (21). Some scholars believe that an unhealthy social environment is one of the incentives to abuse drugs (22). Increased social support can improve cessation outcomes by protecting women from the harmful effects of cessation-related withdrawal symptoms (23). However, whether social support theory is also applicable to the Chinese methamphetamine-dependent population is still not verified.

In China, compulsory detoxification refers to compulsory detoxification treatment, psychological treatment, and legal education for drug addicts through administrative measures within a certain period of time. For people who are seriously addicted to drugs and who are difficult to give up drug addiction through community detoxification, the public security organs can directly make a decision on compulsory isolation and detoxification, and these personnel will enter compulsory detoxification centers for compulsory detoxification and drug addiction treatment. These personnel includes: (1) those who refuse to accept community detoxification; (2) those who take or inject drugs during the period of community detoxification; (3) those who seriously violate the community detoxification agreement; and (4) those who re-take or inject drugs after community detoxification and compulsory isolation. In addition, if drug addicts voluntarily accept compulsory isolation and detoxification, with the consent of the public security organs, they can enter the compulsory detoxification center for detoxification.

Given that the alexithymia, social support, duration of methamphetamine use and their association among male methamphetamine-dependent patients in compulsory detoxification center in China it is not clear. Recent research had highlighted the plasticity of psychological interventions for alexithymia and social support (24–26), which increases the correlation between alexithymia, social support and clinical practice of methamphetamine rehabilitation, and makes relevant research more meaningful. Therefore, we conducted a cross-section survey on male methamphetamine-dependent patients in compulsory detoxification center, to investigated the incidence of alexithymia, social support, as well as the relationship between the duration of methamphetamine use and alexithymia or social support.

## Materials and Methods

### Participants

A total of 137 patients were eligible to participate in the study. Of the 24 excluded samples, eight patients have also used heroin and 16 patients were refused to participate because they did not want to reveal their personal information about illegal drug use. Finally, 113 male methamphetamine-dependent patients were recruited from a men's compulsory detoxification center in Hefei, China. This study was conducted from September to November 2019. Each participant who agreed to participate in the study must sign a formal informed consent form, and the participant had the right to quit at any time. This study was approved by the Ethics Committee of Chaohu Hospital affiliated to Anhui Medical University (201901-kyxm-02). Using medians as cut-off points when no official cut-off points are available is common practice in many studies (27, 28). Patients were categorized by taking the median value (8 years) as the cut-off point for the duration of methamphetamine use.

The inclusion criteria included: (1) meeting the methamphetamine dependence criteria on Diagnostic Interview of the Structured Clinical Interview for DSM-IV (29) conducted by two clinical psychiatrists; (2) non-injecting drug user; (3) aged from 18 to 60 years, Han nationality; (4) provided formal informed consent.

The exclusion criteria included: (1) with serious mental diseases, such as schizophrenia, depression disorder, bipolar disorder, mental retardation et al.; (2) received any psychoactive substance in the past month; (3) with serious physical diseases (including cardiovascular disease, cerebrovascular disease, and neurodegenerative disease et al.); (4) reported multiple illegal drugs of abuse.

### Measures

Socio-demographic data of the participants were assessed by trained research investigators. In our study, duration of dependency was acquired based on the patient's self-report, i.e., time of last drug use minus time of first drug use. We employed the Toronto Alexithymia Scale and Social Support Rating Scale to obtain a comprehensive measure of alexithymia and social support. The uniform instructions used in the test were given by the trained investigators at the beginning of the investigation. During the investigation, for patients who were illiterate or had doubts about the question, investigators would tell him questions and answer options, or explain questions and answer options in the way he could understand, ensuring that patients correctly understood the meaning of each question or answer. All the investigators were psychiatrists from Anhui Medical University.

### Psychiatric Comorbidities

The Semi-Structured Clinical Interview for Axis-I and Axis-II Disorders of the DSM-IV were used to assess patients' psychiatric comorbidities (29).

### Toronto Alexithymia Scale (TAS-20)

Toronto Alexithymia Scale is the gold-standard measure of alexithymia. The scale was compiled by Bagby et al. (30, 31), which contains 20 items and all the items were scored according to the 5-point Likert scale (1 = very disagree, 5 = very agree). It assesses the following three core components: Difficulties identifying feelings (DIF); Difficulties describing feelings (DDF); Externally oriented thinking (EOT). The total score ranges from 20 to 100. The higher the total score is, the higher the alexithymia level is. Individuals with a total score of ≥ 61 were considered to have alexithymia personality traits. TAS is a reliable, valid measure of alexithymia for substance abusers (32). The Chinese version of the scale showed good reliability and validity in domestic studies (33). In this study, the Cronbach' α coefficient of the scale is 0.79, the Cronbach' α coefficient of the DIF, DDF, EOT scale was 0.70, 0.75, 0.63, respectively.

### Social Support Rating Scale

Social Support Rating Scale (SSRS) (34) was employed to assessed the participants' social support. The scale includes 10 items in three dimensions: subjective support, objective support, and support availability. The higher the score of the participants, the higher the level of social support they received. The Chinese version of the Social Support Rating Scale has proved its reliability and validity in the Chinese study (35). In this study, the Cronbach's α coefficient is 0.75.

### Statistical Analysis

Shapiro-Wilk's normality test was performed to access normal distribution. The demographic and clinical data in this study were normally distributed. A chi-square test and *t*-test (Shapiro-Wilk test) were used to investigate categorical variables for demographic data concerning short-duration group and long-duration group (defined using the cut-off scores of 8 years of methamphetamine use). The group differences (short vs. long-duration group) were tested using analyses of covariance (ANCOVA) with short/long-duration as a factor and age, education level, income level, average frequency of methamphetamine use as covariates. Then Pearson correlation analysis and partial correlation controlling with age were used to explore the relationship between the total duration of methamphetamine and scale parameters. Finally, multiple linear regression was used to analyze the related factors of the total duration of methamphetamine abuse. *p* < 0.05 were deemed statistically significant. All statistical testing was two-tailed.

## Results

### Demographic Data and Characteristics of Methamphetamine-Dependent Patients

This study was conducted in the male compulsory detoxification center, therefore all 113 participates were male. The average age and age at onset of methamphetamine use were 30.45 ± 3.81 and 22.40 ± 4.45 years, respectively. The mean duration of methamphetamine use was 8.05 ± 3.84. The mean duration of education was 7.87 ± 3.16 years. A total of 83 males (73.5%) had an education level of junior high school or below, and 113 (26.5%) have an education level of senior high school or above. A total of 47 (41.6%) males were unmarried, 37 (32.7%) males were married, and 29 (25.7%) males were divorced. The number of people who take methamphetamine more than once a week and less than or equal to once a week are 61 (54%) and 52 (46%), respectively. The mean Abstinence duration was 6.21 ± 3.59 months. A cut-off point of 8 (Eight years is the median duration of methamphetamine use in this study) was used to divide participants into long-duration group (*n* = 59) and short-duration group (*n* = 54).

### Demographic Characteristics Between Short-Duration and Long-Duration Patients

Table 1 summarizes the differences in personal characteristics between short-duration and long-duration use of methamphetamine-dependent patients. Among them, the differences in marital status, education level, frequency of methamphetamine use, and income level were not statistically significant (all *p* > 0.05). However, significant differences were observed in age (29.53 ± 3.79 vs. 31.46 ± 3.60; *t* = −2.78, *P* = 0.006), and age at onset of methamphetamine use (24.56 ± 4.11 vs. 20.04 ± 3.54; *t* = 6.24, *P* < 0.001).

**Table 1:** Comparison of the demographic characteristics between short-duration and long-duration groups.

**Variables**	**Total sample (*n* = 113)**	**Short-duration group (≤8 years, *n* = 59)**	**Long-duration group (>8 years, *n* = 54)**	***X*^**2**^/*t***	***P***
Age (years)	30.45 ± 3.81	29.53 ± 3.79	31.46 ± 3.60	−2.78	0.006
Age at onset of MA use (years)	22.40 ± 4.45	24.56 ± 4.11	20.04 ± 3.54	6.24	<0.001
Duration of MA use (years)	8.05 ± 3.84	4.97 ± 2.11	11.43 ± 2.01	−6.46	<0.001
Abstinence duration (months)	6.21 ± 3.59	5.76 ± 3.33	6.70 ± 3.78	−1.23	0.223
Marital status				2.30	0.316
Single	47 (41.6%)	23 (39%)	24 (44.4%)		
Married	37 (32.7%)	23 (39%)	14 (25.9%)		
Divorced	29 (25.7%)	13 (22%)	16 (29.6%)		
Education level				0.33	0.569
Junior middle school or below	83 (73.5%)	42 (71.2%)	41 (75.9%)		
Senior high school	30 (26.5%)	17 (28.8%)	13 (24.1%)		
Frequency of MA use				1.16	0.282
>1 time/week	61 (54%)	29 (49.2%)	32 (59.3%)		
≤1 time/week	52 (46%)	30 (50.8%)	22 (40.7%)		
Income level				0.07	0.797
<3,000	26 (23%)	13 (22%)	13 (24.1%)		
≥3,000	87 (77%)	46 (78%)	41 (75.9%)		

*MA, Methamphetamine*.

### Alexithymia and Social Support Between Short-Duration and Long-Duration Patients

The prevalence of alexithymia in male methamphetamine-dependent patients in compulsory detoxification was 23% (26/113) in the whole sample group with rates of 13.6% (8/59) in short-duration group and 33.3% (18/54) in long-duration group, respectively. Significant differences were observed between groups (13.6 vs. 33.3%, χ^2^ = 6.22, df = 1, *P* = 0.013) (Table 2). Alexithymia and social support of patients are shown in Table 2. The long-duration patients had significantly lower social support levels, subjective support levels, support availability levels (all *p* < 0.05), but higher alexithymia ratio (33.3 vs. 13.6%, *p* < 0.05), alexithymia level and difficulties identifying feelings levels (all *p* < 0.05) as compared to short-duration patients. Effect size calculations (η^2^) showed that the magnitude of the group effect for the social support score (0.07), subjective support (0.10), and DIF (0.06) was moderate. Since age, education level, income level, average frequency of methamphetamine use were significant factors associated with duration of methamphetamine use, we ran an ANOVA by using age, education level, income level, average frequency of methamphetamine use as covariates. After controlling the effects of the above covariables, significant differences were also found in social support total score (*F* = 3.18, *p* < 0.05), between short and long duration groups. Effect size calculations (η^2^) showed that the magnitude of the group effect for the social support score (0.12) was moderate. However, there is no significant difference in subjective support, support availability, TAS total score and difficulties identifying feelings between short and long-duration patients (all *p* > 0.05).

**Table 2:** Comparison of social support rating scale and twenty–item Toronto alexithymia scale scores between short duration and long duration groups (Mean ± SD).

**Variables**	**Short duration group (≤8 years)**	**Long duration group (>8 years)**	**Before adjustment**			After adjustment^*^		
			***F*/χ^2^**	***P***	**η^2^/ϕ**	***F***	***P***	**η^2^**
SS total score	36.92 ± 7.80	33.09 ± 5.49	3.03	0.003	0.07	14.29	<0.001	0.12
Subjective support	22.47 ± 5.19	19.31 ± 4.58	3.42	0.001	0.10	1.58	0.211	0.02
Objective support	7.71 ± 3.01	7.80 ± 2.59	−0.16	0.874	<0.01	1.79	0.195	0.02
Support availability	6.73 ± 1.86	5.98 ± 1.54	2.32	0.022	0.05	0.42	0.516	0.01
Alexithymia			6.22	0.013	0.23			
NO *n* (%)	51 (86.4)	36 (66.7)						
YES *n* (%)	8 (13.6)	18 (33.3)						
TAS total score	52.95 ± 7.75	56.43 ± 8.13	5.42	0.022	0.05	0.61	0.437	0.01
DIF	17.73 ± 4.35	20.06 ± 4.76	7.38	0.008	0.06	0.96	0.330	0.01
DDF	13.95 ± 2.22	14.35 ± 3.02	0.66	0.418	0.01	2.31	0.131	0.02
EOT	21.27 ± 4.04	22.02 ± 2.79	1.29	0.259	0.01	3.67	0.058	0.03

*TAS, Toronto alexithymia scale; DIF, Difficulties identifying feelings; DDF, Difficulties describing feelings; EOT, Externally oriented thinking; SS, Social support*.

* *Adjusted for age, education level, income level, average frequency of methamphetamine use*.

### Correlation of Duration of Methamphetamine Use, Alexithymia, and Social Support

Correlation analysis showed that significant correlations between duration of methamphetamine use and the following parameters: difficulties identifying feelings score (*r* = 0.24, df = 113, *p* < 0.05), social support total score (*r* = −0.30, df = 113, *p* < 0.01), subjective support (*r* = −0.30, df = 113, *p* < 0.01), support utilization (*r* = −0.25, df = 113, *p* < 0.01) (Table 3).

**Table 3:** Correlation matrix of methamphetamine use duration, social support rating scale and twenty–item Toronto alexithymia scale scores of the methamphetamine-dependent patients (Mean ± SD).

**Variable**	**Mean SD**	**Duration of MA use**	
		**Pearson correlation**	**Partial correlation controlling with age**
Duration of MA use	8.05 ± 3.84	1.00	1.00
TAS total score	54.61 ± 8.09	0.18	0.21^*^
DIF	18.84 ± 4.67	0.24^*^	0.27^**^
DDF	14.14± 2.63	0.08	0.08
EOT	21.63 ± 3.50	0.03	0.06
SS total	35.09 ± 7.03	−0.30^**^	−0.33^***^
Subjective support	20.96 ± 5.14	−0.30^**^	−0.35^***^
Objective support	7.75 ± 2.81	−0.04	−0.04
Support availability	6.37 ± 1.74	−0.25^**^	−0.21^*^

* *P < 0.05*,

** *P < 0.01*,

*** *P < 0.001; MA, Methamphetamine; TAS, Toronto alexithymia scale; DIF, Difficulties identifying feelings; DDF, Difficulties describing feelings; EOT, Externally oriented thinking; SS, Social support*.

Furthermore, multiple linear regression was used to analyze the risk factors of methamphetamine use time in methamphetamine-dependent patients. The results of the multi-factor logistic model are shown in Table 4. It has been verified that the observations in the study were independent of each other (Durbin-Watson test value = 1.77), and the regression model was statistically significant (*F* = 6.52, p < 0.001, *R*^2^ = 0.30, Adjusted *R*^2^ = 0.26). In the multiple Logistic regression analysis related to the duration of methamphetamine use, the effects of three independent variables (age, difficulties identifying feelings, subjective support) on the duration of disease were statistically significant (*P* < 0.05).

**Table 4:** Predictors generated by Multivariate Logistic Regression with duration of methamphetamine use as dependent variables.

	**Unstandardized coefficients**		**Standardized coefficients**	**t**	***p***	**95.0% CI**	
	**B**	**S.E**	**Beta**			**LB**	**UB**
(Constant)	0.22	4.06		0.05	0.96	−7.84	8.27
Age	0.36	0.08	0.36	4.27	<0.001	0.19	0.53
DIF	0.24	0.09	0.29	2.81	0.01	0.07	0.41
DDF	−0.21	0.15	−0.15	−1.40	0.17	−0.51	0.09
EOT	0.08	0.09	0.07	0.83	0.41	−0.11	0.26
Subjective social support	−0.24	0.06	−0.33	−3.79	<0.001	−0.37	−0.12
Objective social support	0.08	0.12	0.06	0.70	0.49	−0.15	0.32
Support availability	−0.29	0.19	−0.13	−1.49	0.14	−0.67	0.10

*DIF, Difficulties identifying feelings; DDF, Difficulties describing feelings; EOT, Externally oriented thinking*.

## Discussion

To the best of our knowledge, our study is the first to explore the association between alexithymia characteristics, social support characteristics, and duration of methamphetamine use among male methamphetamine-dependent patients in Chinese compulsory detoxification center. The main conclusions of this study are as follows: (1) Twenty-three percent of males in compulsory detoxification centers were considered to have alexithymia personality traits, which is significantly higher than the incidence in the general population (36, 37). (2) Difficulties identifying feelings level is significantly positively related to the duration of methamphetamine use. (3) Subjective social support is significantly negatively related to the duration of methamphetamine use.

Previous studies have investigated the incidence of alexithymia in people with different cultural backgrounds (36–38). However, the incidence of alexithymia in the Chinese male compulsory detoxification population of methamphetamine is still unclear. We found that the incidence of alexithymia in the Chinese male compulsory detoxification population of methamphetamine was 23% (26/113) in the whole sample group with rates of 13.6% (8/59) in the short-duration group and 33.3% (18/54) in the long-duration group. Considering that the incidence of alexithymia in the Chinese general population is only 16.67% (39), it seems that the incidence of alexithymia among Chinese male methamphetamine-dependent patients, in general, is higher than in the general population. The findings are consistent with other studies of substance addicts in Western countries, such as a U.S. study that showed a high percentage (50.4%) of mixed substance abusers (drugs and alcohol, etc.) scored in the alexithymia range (32). A British study indicated that 46% of opiate addicts scored in the alexithymia range (40). Interestingly, the incidence of alexithymia in the substance-dependent population in the above study was higher than that in our study, which may be related to the type of substance dependence.

Our results suggest a potential relationship between difficulties identifying feelings levels and maintenance and relapse of methamphetamine use. Long-duration methamphetamine users had significantly higher difficulties identifying feelings levels than short-duration methamphetamine users. After adjusting for confounding factors, only difficulties identifying feelings levels in the three dimensions of alexithymia were significantly associated with the duration of methamphetamine use. That is, methamphetamine addicts with higher difficulties identifying feelings had a higher risk of continued methamphetamine use. However, difficulties describing feelings and externally oriented thinking are not significantly related to methamphetamine use duration. Previous research on emotion regulation supports our view that being aware of feelings and accurately identifying feelings are important for effective emotion regulation (41, 42), people with alexithymia who cannot accurately identify emotions often use ineffective emotion regulation methods (43, 44), such as abusing drugs. These conditions may make people with alexithymia traits unable to effectively regulate their negative emotions in the face of stress, leading to the long-term persistence of negative feelings (9). Methamphetamine users experience additional stress and mental symptoms in many areas of their lives (45, 46). Previous research has shown that relieving bad emotions is one of the important motivations for drug use (47, 48). Drug users report that they choose to use drugs due to loneliness, emptiness and pain (47, 48). For example, a survey showed the greatest number of respondents (56%) affirmed that they used drugs due to “pleasure-seeking,” while 30% of respondents choose to take drugs or relapse because of “Pain Avoidance” (47). In conclusion, when having negative feelings, people with alexithymia personality traits tend to use negative coping strategies to identify and deal with negative feelings and are more likely to continue to choose methamphetamine to escape or alleviate negative feelings, leading to the continued use or relapse of methamphetamine, and further increasing the duration of drug use. A similar pattern appears in other substance dependent people, such as those with high levels of alexithymia, who use high-risk drinking behavior to regulate their emotions and avoid negative emotional feelings (49). De Berardis et al. explain that alexithymia can lead to emotional disturbances and dysregulation in adolescents that further increase the risk of addictive behaviors and suicidal thoughts in impending adult life, exacerbating emotional dysregulation, forming a vicious cycle that will be difficult to break (13, 15, 19, 20). Higher alexithymia scores were related to higher levels of depression, anxiety, impulsivity in patients who had cannabis use disorder. Not surprisingly, depression, anxiety, and impulsivity aggravate the risk of continued drug use (16).

Our study suggests a potential relationship between subjective social support and maintenance and relapse of methamphetamine use. Patients with long-duration methamphetamine dependence reported lower subjective social support than those with short-duration methamphetamine dependence. After adjusting for confounding factors, higher subjective social support was significantly associated with a lower duration of methamphetamine use, which is consistent with previous studies. For example, Chinese patients receiving methadone maintenance treatment with poorer perceived social support were more likely to terminate treatment, with hazard ratios of 1.31 (without controlling baseline information and past retention) and 1.25 (controlling baseline information and past retention) (50). Social environment of people in compulsory detoxification affects his therapy outcome, extra-institutional support was highly associated with relapse (8). Improving social support for drug abusers may be fruitful in eliminating the negative effects of internal stigma on mental health and well-being, and is likely to increase the success rate of drug withdrawal (51). In our study, we conducted in-depth interviews with 10 interviewees and professors in the compulsory drug rehabilitation center. The results of the interviews show that social acceptance and tolerance of drug addicts in China are very low. When the methamphetamine-taking behavior is known by family members, relatives and friends, it is very common for the methamphetamine user to be alienated, blamed and rejected, and the subjective social support they feel is seriously reduced. Rehabilitated methamphetamine abusers think it is difficult for them to be accepted by their original circle of friends, and it is also difficult for them to integrate into the original circle of friends again. The vast majority of methamphetamine rehabilitation patients lose confidence in themselves and their ability to reintegrate into society, and they have to return to the drug circle because they can find a sense of existence and common topics among their peers who use methamphetamine. When methamphetamine users see their peers taking methamphetamine or hear drug-related clues, they are stimulated to produce conditioned reflexes, which leads to a particular desire to use methamphetamine. This will cause the addicts to be exposed to methamphetamine again, which is the biggest cause of relapse.

This study has several limitations. First of all, there was no female that enrolled in this study, because we haven't been allowed to investigate in female's detoxification center. However, a large body of evidence has shown that TAS-20 score and prevalence of alexithymia (with a cut-off of 61) in patients with substance use disorders showed no gender differences (14–16), implying that there is no general effect of sex on the relationship between methamphetamine use and alexithymia or social support. Furthermore, in future work, we will definitely expand the sample size to include larger more patients including women. Second, as a cross-sectional study, the results of this study do not show the causal relationship between difficulties identifying feelings, subjective social support and methamphetamine abuse duration. Therefore, our main findings in this study should be regarded as preliminary, further study should be done to confirm causality in research results using longitudinal and prospective designs. Third, since all the subjects were recruited from a compulsory detoxification center in Hefei, Anhui Province, China, whether our results can be extended to other parts of China is worthy of further study. Another limitation of this study was the use of self-rating scales.

## Conclusion

This study reports a significant association or “signal” between the duration of methamphetamine use and difficulty in identifying feelings or subjective social support of Chinese male methamphetamine-dependent patients. While the data have limitations that preclude a definitive causal conclusion, this finding should be considered in the psychotherapy of methamphetamine rehabilitation, which indicates that the psychotherapy targeted on subjective social support and difficulty in identifying feelings may be potentially promising treatments for methamphetamine-dependent patients in China. We expect that our findings will arouse people's attention to the potential risks of high difficulty in identifying feelings and low subjective social support for methamphetamine-dependent patients. This provides a clue to our next step of psychological intervention research to prevent relapse among methamphetamine-dependent patients in compulsory detoxification centers.

## Data Availability Statement

The original contributions presented in the study are included in the article/supplementary files, further inquiries can be directed to the corresponding author/s.

## Ethics Statement

This study was approved by the Ethics Committee of Chaohu Hospital affiliated to Anhui Medical University (201901-kyxm-02). The patients/participants provided their written informed consent to participate in this study.

## Author Contributions

XZ: conception and design and provision of study materials or patients. XZ and KZ: administrative support. SC, FC, QY, LZ, and LW: collection and assembly of data. SC and KZ: data analysis and interpretation. All authors: manuscript writing and final approval of manuscript.

## Funding

We thank all of patients who volunteered to participate in the study. This study was supported by the National Natural Science Foundation of China (81801341), the Anhui Provincial Key R&D Programme (202004j07020030).

## Conflict of Interest

The authors declare that the research was conducted in the absence of any commercial or financial relationships that could be construed as a potential conflict of interest.

## Publisher's Note

All claims expressed in this article are solely those of the authors and do not necessarily represent those of their affiliated organizations, or those of the publisher, the editors and the reviewers. Any product that may be evaluated in this article, or claim that may be made by its manufacturer, is not guaranteed or endorsed by the publisher.

## Abbreviations

TAS: Toronto alexithymia scale
DIF: Difficulties identifying feelings
DDF: Difficulties describing feelings
EOT: Externally oriented thinking
SS: Social support.
